# Trends in Socioeconomic Inequalities in the Distribution of Dental Caries Among Brazilian Adolescents, 2003–2023

**DOI:** 10.1590/1980-549720260017.supl.1

**Published:** 2026-07-31

**Authors:** Sophia Queiroz Marques dos Santos, Angelo Giuseppe Roncalli

**Affiliations:** I Universidade Federal do Rio Grande do Norte, Postgraduate Program in Public Health – Natal (RN), Brazil.

**Keywords:** Measuring health inequalities, Oral health, Epidemiological surveys, Dental caries

## Abstract

**Objective:**

Analyze trends in socioeconomic inequalities in the distribution of dental caries among adolescents in the years 2003, 2010, and 2023.

**Methods:**

Longitudinal observational study using data from national oral health surveys. The Decayed, Missing and Filled Teeth (DMFT) index and the number of decayed teeth were analyzed, stratified by income and education. The analyses accounted for the complex sampling design and included measures of absolute inequality (*Slope Index of Inequality* – SII) and relative inequality (*Relative Index of Inequality* – RII). Statistical significance was assessed at the 95% confidence level (p < 0.05).

**Results:**

A global reduction in DMFT (Decayed, Missing, and Filled Teeth) and the number of decayed teeth was observed across all socioeconomic strata throughout the period, indicating a general improvement in oral health conditions. However, marked inequalities persisted between groups, maintaining the social gradient, in which adolescents with lower income and education levels presented worse indicators. The SII (Social Income Index) indicated stability in the absolute differences between the social extremes, while the RII (Inequality Index) showed variations over time, suggesting proportional changes in inequalities. For DMFT, an increase in inequalities was observed according to education level and a slight reduction in relation to income; for missing teeth, there was a reduction in inequalities in the most recent years.

**Conclusion:**

Despite the overall improvement in caries indicators, socioeconomic inequalities in oral health among adolescents persist, reflecting structural social determinants. The findings reinforce the need for public policies aimed at reducing inequities and promoting equity in oral health.

## INTRODUCTION

The social determinants of health reflect the living conditions and social arrangements that influence the development of diseases^1^. These determinants, in turn, function as a specific segment within a larger set, with the aim of identifying how different social groups present distinct health outcomes. The analysis of inequalities reveals the social gradient of health, a hierarchical pattern in the distribution of diseases, which suggests that variations in socioeconomic status directly impact susceptibility to health conditions^2^.

To illustrate this phenomenon, we can consider Food and Nutritional Insecurity (FNI), traditionally associated with malnutrition. Today, after significant changes in the market and in “social practices”—as conceptualized by Max Weber—overweight and obesity have become new markers of vulnerability, broadening the concept of FNI, which now also encompasses forms of undernutrition^3^. Despite behavioral transformations and the introduction of new perspectives for the analysis of health problems, the social gradient on the outcome is so strongly influential that specific populations continue to experience the worst health conditions. A similar situation is observed in oral health.

In the context of oral health, a crucial topic is the cost of ensuring the effectiveness of services in the public health system. We are referring not only to the funding required for the treatment of already existing lesions, but also to the forecast of expenses related to imminent lesions. The underfunding of the system directly contributes to the perpetuation of inequalities and reveals important social markers, since the cost of treatment for individuals in lower socioeconomic conditions is related to historical and social neglect. This results in a higher prevalence or worsening of lesions, increasing costs—still not fully covered^4,5^. In practice, equal access to oral health is a challenge, as various individual, organizational, and social factors hinder the fulfillment of this principle^6^. The development of the disease often exceeds the capacity for treatment, which progresses slowly.

In a study conducted in 2015 by Roncalli et al., it was observed that the decline in dental caries among Brazilian adolescents between 2003 and 2010 occurred unevenly and that the inequality indicators analyzed in these two periods pointed to a worsening of socioeconomic differences^7^. Similarly, a comparative investigation of the National School Health Survey (PeNSE) 2009, 2012, and 2015 showed that the prevalence of toothache increased from 17.5% to 21.8% between the extreme years, with adolescents whose mothers had lower levels of education consistently presenting higher levels of toothache^8^. A more recent analysis of 12-year-old adolescents in 2003, 2010, and 2023 indicated an overall reduction in caries experience, however with persistence of regional inequalities, where the worst stratum had an outcome about four times higher than the average^9^.

The polarization in the distribution of oral diseases is not a recent phenomenon; nevertheless, its continuous monitoring is essential to identify distribution patterns and support strategies that are truly capable of mitigating the persistence of inequality. In this sense, this study seeks to continue the investigation of trends in socioeconomic inequalities in the distribution of caries among adolescents between the years 2003, 2010, and 2023.

## METHODS

This is a longitudinal observational study, developed with secondary data from the three most recent national oral health surveys, known as the SB Brasil Project (2003, 2010, and 2023)^10–12^, approved by the respective Research Ethics Committees (processes 25000.009632/00-51, No. 1.356, No. 15.498, and No. 4.823.054). The surveys used similar methodologies, without compromising the representativeness and comparability of the data. The main outcomes investigated included dental caries, periodontal condition, malocclusions, and the need for and use of dental prostheses. Detailed information on the methodology of the SB Brasil Project can be found in specific publications, especially in the works of Roncalli & Souza^13,14^.

The sample was composed of adolescents aged 15 to 19 years, totaling 16,833 individuals in 2003, 5,445 in 2010, and 8,054 in 2023. This age group was selected due to the availability of individual socioeconomic data and the lower variability in caries rates, favoring association analyses.

### Variables

The outcomes were the Decayed, Missing and Filled Teeth index (DMFT) and the number of decayed teeth (component “C”). The independent variables were monthly family income and educational level, available in the questionnaires from the three surveys. Income was measured as total household income in the month prior to the interview, recorded in absolute values in 2003 and 2023, and in categories in 2010. For standardization, income was converted into minimum wages in effect in each year (2003: R$ 210; 2010: R$ 510; 2023: R$ 1,320) and categorized into 4 strata.

Schooling was expressed in years of study and adjusted for age, using the age-grade distortion indicator, widely used in the evaluation of educational policies. The variable was categorized into four strata (above the ideal level, at the ideal level, 1–2 years behind, and ≥ 3 years behind), following the strategy adopted by Roncalli et al.^7^.

### Data analysis

Initially, a descriptive analysis of the mean DMFT index and the number of decayed teeth was performed, with their respective 95% confidence intervals, according to socioeconomic strata and survey years (2003, 2010, and 2023). Considering that the three surveys used a complex sampling design^14^, all estimates incorporated sampling weights and design effects, including the Primary Sampling Units (PSU) as *clusters*.

### Conceptualization and interpretation of inequality indices

The inequality in the distribution of dental caries was measured using the *Slope Index of Inequality* (SII) and the *Relative Index of Inequality* (RII), calculated for each outcome, socioeconomic variable, and year analyzed. The interaction effect between the surveys was also estimated to assess changes in inequality indicators between 2003, 2010, and 2023.

The SII and RII were estimated using Generalized Linear Models (GLM) with a Poisson distribution, as recommended by Mackenbach & Kunst (1997)^15^ and Barros & Victora (2013)^16^. The independent variables were transformed into ridit scores, based on the proportional distribution of the sample in each category. The SII represents the absolute difference between the extremes of the social hierarchy, while the RII expresses the relative ratio between these groups. The analyses considered sample weights and *cluster* effect using the *svy* command in *Stata* software.

*Equiplot* graphs were used to illustrate inequalities, according to the methodology proposed by the International Center for Equity in Health, Universidade Federal de Pelotas (UFPel), Brazil. Visually, greater distances between points along the socioeconomic scale indicate a greater magnitude of inequality (https://equidade.org/pt/home).

### Data availability statement

The entire dataset that supports the results of this study was made available in SciELO Data (https://doi.org/10.48331/SCIELODATA.HINAG2) and can be accessed at https://www.gov.br/saude/pt-br/composicao/saps/brasil-sorridente/sb-brasil/dados.

## RESULTS

**Figure 1:**
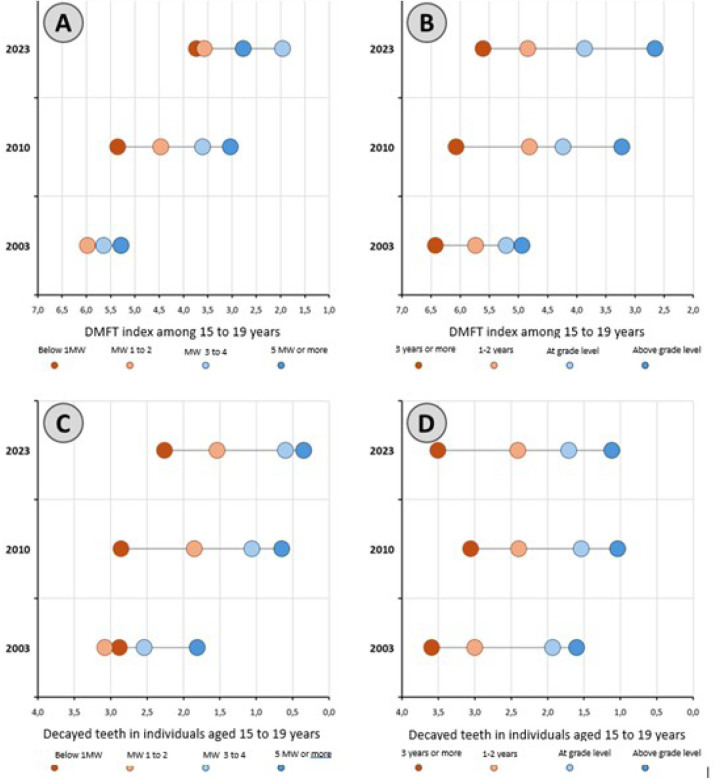
Equiplots illustrating the distribution of the DMFT index in relation to income (A) and educational level (B), and the number of decayed teeth in relation to income (C) and educational level (D), for the three years studied. Source: Prepared by the authors based on data from the SB Brasil 2003, 2010, and 2023 databases.

Table 1 and Figure 1 present the results of the descriptive analysis comparing the DMFT index and the number of decayed teeth for each stratum of socioeconomic variables (income and educational level) and for the years studied.

**Table 1:** Mean DMFT (absolute value) and mean number of decayed teeth (relative value to the overall mean) in adolescents aged 15–19 years, according to socioeconomic variables and year of the survey.

	n	DMFT		Decayed teeth	
		Average	95%CI	Average	95%CI
Family income (minimum wages – MW)					
2003					
More than 5 minimum wages	5,298	5.29	4.85–5.74	1.81	1.55–2.08
3 to 5 MW	3,724	5.65	5.19–6.10	2.54	2.28–2.80
1 to 2 MW	3,546	5.98	5.47–6.50	3.08	2.78–3.37
Less than 1 MW	3,955	5.29	4.9–5.68	2.88	2.56–3.2
2010					
Greater than 5 MW	665	3.04	2.36–3.72	0.65	0.44–0.86
3 to 5 MW	920	3.61	2.99–4.24	1.06	0.80–1.32
1 to 2 MW	2.621	4.47	4.04–4.91	1.85	1.55–2.15
Less than 1 MW	846	5.36	4.51–6.21	2.86	2.19–3.52
2023					
Greater than 5 MW	343	2.77	1.45–4.09	0.35	0.16–0.53
3 to 5 MW	689	1.96	1.15–2.76	0.60	0.33–0.86
1 to 2 MW	2.563	3,57	3.01–4.12	1.54	1.25–1.82
Less than 1 MW	1.416	3,74	3.15–4.33	2.26	1.86–2.66
Educational level (age-grade distortion)					
2003					
Above	3,054	4.94	4.49–5.39	1.60	1.34–1.85
Ideal level	5,451	5.21	4.79–5.63	1.93	1.69–2.17
1-2 years	3,509	5.74	5.32–6.16	3.00	2.72–3.28
3 or more years	4,008	6.43	6.03–6.84	3.59	3.26–3.91
2010					
Above	1,398	3.23	2.76–3.70	1.04	0.79–1.28
Ideal level	2,044	4.24	3.66–4.82	1.54	1.25–1.84
1-2 years	926	4.81	4.08–5.53	2.40	1.09–2.90
3 or more years	662	6.07	5.24–6.89	3.06	2.44–3.69
2023					
Above	3,390	2.66	2.20–3.12	1.12	0.88–1.35
Ideal level	3,507	3.87	3.40–4.35	1.71	1.46–1.95
1–2 years	508	4.84	3.90–5.77	2.41	1.74–3.08
3 or more years	200	5.61	4.11–7.11	3.51	2.44–4.58

A reduction in the average DMFT index is observed between 2003 and 2023 in all income and educational level strata. In the analysis of the degree of inequality, a gradient is expected in which the most favored strata will always have more favorable values, which worsen as the less favored strata also worsen, expressing a dose-response effect.

This pattern is present in virtually all analyses, except for DMFT in relation to income in 2003 (same values for the first and fourth strata) and for the number of decayed teeth in relation to income in the same year (inversion between the first and second strata). However, in both cases, the values are close and there is no significant difference, according to the analysis of the confidence intervals.

It can be inferred that there is a dose-response effect for DMFT (Decayed, Missing, Filled Teeth) and decayed teeth in relation to income and educational level, expressing, in practice, the inequality in the distribution of the disease. However, the magnitude of this effect and whether it changes over the years should be observed. In the visual analysis, it is observed whether the distance between the circles changes between the years studied. For DMFT, there is a greater deepening of inequality between 2003 and 2010, followed by stability between 2010 and 2023, both for income and educational level. For decayed teeth, the profile is similar, with the exception of the relationship with educational level, which shows a pattern of inequality that tends towards improvement, but is relatively similar between the years.

Overall, the analysis of inequalities in oral health revealed that, for all outcomes evaluated, marked differences persist between the extremes of income and education, evidenced by the SII and RII indices. The SII, measured by the difference in outcome between the most favored and least favored groups, showed little statistically significant variation over the period, indicating that, in absolute terms, the distance between the extremes remained stable. In contrast, the RII, which expresses the ratio between the extremes, showed significant changes in several cases, suggesting that the proportional strength of inequality has changed over the years (Table 2).

**Table 2:** Absolute inequality index (SII) and relative inequality index (RII) for the mean DMFT index and decayed teeth. The p-value indicates the presence of significant variations between the years.

	SII (95%CI)				RII (95%CI)			
	2003	2010	2023	p-value	2003	2010	2023	p-value
DMFT								
Family income (minimum wages)	0.38 (-0.42–1.17)	2.66 (1.57–3.75)	1.91 (0.41–3.40)	**0.051**	1.06 (0.92–1.22)	1.87 (1.44–2.42)	1.70 (1.10–2.61)	**0.006**
Education (age-grade distortion)	1.46 (0.86–2.07)	2.17 (1.20–3.14)	2.26 (1.25–3.26)	0.301	1.31 (1.17–1.48)	1.67 (1.34–2.09)	1.79 (1.38–2.33)	**0.038**
Decayed teeth								
Family income (minimum wages)	1.86 (1.35–2.37)	2.48 (1.84–3.11)	2.42 (1.86–2.98)	0.163	2.04 (1.68–2.48)	4.67 (3.16–6.92)	4.49 (2.92–6.94)	**<0.001**
Education (age-grade distortion)	2.14 (1.7–2.52)	1.52 (1.00–2.03)	1.09 (0.52–1.66)	0.209	2.69 (2.19–3.31)	2.51 (1.81–3.48)	1.91 (1.37–2.64)	**<0.001**

In the case of the RII for DMFT, an initial increase is observed, followed by a reduction in income-related inequalities. However, the analysis according to education level indicates a growing gap between the groups, highlighting distinct impacts of social factors on oral health. Regarding the number of decayed teeth, the behavior follows a pattern similar to income, with a tendency for inequalities to decrease over time (Table 2). This suggests that, although adolescents have increased access to dental services and reduced the number of decayed teeth, these advances are not yet reflected in an effective decrease in caries experience.

## DISCUSSION

An uneven change in the disease pattern can be observed, with a mismatch between the decline in the average number of decayed teeth and the DMFT index. The continuity of caries experience shows a slow regression, with a pattern of decreasing decayed teeth over time, more pronounced in more affluent groups. The distance between these groups is mainly influenced by income and educational attainment. The absolute inequality index did not show a significant difference between the three periods, indicating that inequality persists over time.

However, the relative index showed a significant change, highlighting the differences in the probability of becoming ill. In 2003, caries experience in the lowest income group was 6% higher than in the highest income group, increasing significantly to 87% in 2010 and decreasing slightly to 70% in 2023 (p=0.006), evidencing a persistent inequality – and a similar trend occurs for decayed teeth. For educational level, caries experience in the least favored group was 31% in 2003, increasing to 67% in 2010 and 79% in 2023 (p=0.038); the inequality between educational levels intensified in the DMFT index, but the same trend is not observed for decayed teeth, with a decrease over the years. These results are fundamental to guiding the formulation of strategies within the scope of the National Oral Health Policy (PNSB), focusing on the studied population, aiming at reducing inequalities in oral health.

Excerpts regarding the socially unequal distribution of chronic diseases are common; however, comparisons over time are rarely available, especially for oral diseases. A study that associated individual behaviors with the development of these diseases revealed that, regardless of health practices, a gap existed between groups, resulting from deeper social factors^17^. Complementarily, a cost analysis of oral treatments in six countries (Brazil, France, Germany, Indonesia, Italy, and the United Kingdom) revealed that, for most of them, the highest cost was concentrated in the most vulnerable social stratum of the population, except for Indonesia^18^, reaffirming a heterogeneous and socially graduated distribution.

These findings reveal that societies with different behaviors, health systems, and living conditions still reflect the distributional arrangement of disease at different levels. Seeking to detail the local scenario, the research condenses a socioeconomic questionnaire with clinical findings, from which it was possible to extract variables such as income and education, essential in creating a model of determination. Furthermore, the evolution of policies and their expression in society are extremely relevant to the research results, composing the last level of a model of determination. Achievements related to social rights began to develop in Brazil in the 1990s, while the 2000s were marked by attempts at operationalization, and subsequent decades symbolize greater effectiveness of the service, justifying the significant improvement in oral health seen in the data from 2003 and 2010^19^.

The discrepancies between subsequent years, besides being considerably smaller, do not always result in a reduction. The evaluation of these services may be one of the key elements to justify this scenario. The PNSB, which turned 20 years old in 2024, was fundamental in the attempt to increase coverage and improve access to oral health^20^. Furthermore, there are disparities in the use of services, considering that the black population of the North and Northeast regions, with low economic and educational conditions, has a greater chance of irregular dental care and of undergoing surgical or emergency procedures^21^, indicating that the implementation of the policy was not sufficient to mitigate inequalities in access to dental consultations^22^. The study by Freire et al. demonstrated that “non-access” was frequently associated with municipal factors, such as greater inequality, organizational factors, such as lower coverage and travel time to the unit, in addition to individual factors such as age and income^23^.

The problem, however, may extend to the distribution of services and the availability of professionals. It is possible to link the higher prevalence of oral health teams to cities with a very high Human Development Index (HDI), located in the Southeast region and with coverage of less than 50%^24^, while the workforce also shows a higher proportion per 10,000 inhabitants in the South and Southeast regions^25^. Although public oral health policies improve the situation, they have not eliminated inequalities in access and care, especially among the most vulnerable groups, something that is clearly evidenced by the study results.

It is also important to reflect on how age range and historical contexts influence the results. Considering that 5-year-old children in 2010 were 18 years old in 2023, it is noteworthy that they were born in a time marked by the implementation of the PNSB (National Oral Health Policy) and experienced a completely different reality compared to 18-year-old children in 2010, who at 11 (2003) years old only suffered the impact of the social gradient. It is undeniable that public policy, to some extent, mitigates inequalities for all groups, but in different ways^26^. Younger children, even those born after the implementation of the PNSB, still face significant social barriers when they belong to lower socioeconomic strata – challenges that often exceed the effectiveness of the policy^27^. Thus, the reduction of oral diseases does not occur homogeneously among groups. In the most vulnerable, even with public policies, advances in oral health tend to be slower and more unequal; and the opposite is also true. Paradoxically, this could end up widening existing disparities instead of reducing them.

The significant impact of determinants such as education and income on oral health has been extensively studied in the international literature^28,29^. Individuals with lower educational levels tend to place less value on preventive oral health practices, which, coupled with a lack of access to adequate information, increases the prevalence of caries and other oral diseases^28^. Similarly, lower income is directly related to difficulty accessing quality oral health services, including regular check-ups and preventive treatments, and is frequently associated with diets richer in sugars and processed foods, contributing to the worsening of oral diseases^29^. Nevertheless, the interaction of these determinants exacerbates the disease scenario.

Although schooling is often associated with greater access to information and preventive behaviors, as reflected in the Social Embodiment Theory^30^, which emphasizes socially determined behavioral repetitions, its relationship with cumulative indicators such as DMFT is more complex, something clearly captured in this study. Changes in educational level or the quality of formal education accessed do not necessarily translate into an immediate reduction in accumulated caries experience, since this indicator reflects exposures and care patterns built up over the course of life. In this sense, schooling acts as a structural marker of social trajectories and historically unequal access to oral health services, influencing the type of care received (preventive or mutilating) more strongly than the current course of disease activity. This is strongly related to Life Course Epidemiology^31^, suggesting that initial exposures, whether positive or negative, reverberate in the individual over the years. The above helps explain why the reduction in inequalities was more evident according to income and the active component of the disease, while inequalities related to education persisted and intensified in the DMFT index.

In countries like the United Kingdom, with public policies such as the National Health Service (NHS), there have been important advances in reducing inequalities in oral health, offering free or affordable treatments for different age groups. However, even with these policies, significant disparities in oral health outcomes persist among different socioeconomic groups^32^. A study conducted by the World Health Organization (WHO) in 2017 highlights that, despite prevention efforts, proximal determinants remain the strongest predictors of oral health conditions, with the most disadvantaged populations, especially those with low income and education levels, suffering more acutely from oral diseases^33^.

The multifactorial nature of oral diseases demands an integrated and comprehensive response from public policies, which must consider the complexity of social and economic conditions^34^. To effectively combat inequalities, it is necessary to go beyond the promotion of care (epidemiologically determined, properly funded, and intersectorally integrated), extending to the advancement and integration of other public policies. Oral health policies often act in isolation, without transversality, but when combined with basic sanitation and food policies, the effects can be multiplied, potentially being more effective in a population where sanitary conditions are adequate and dietary habits are more balanced^35^. Strengthening these policies simultaneously would help create a more favorable environment for oral health. As mentioned earlier, the period from 2000 to 2010 was marked by a focus on public health policies and poverty reduction in Brazil^36^, which may have contributed to the marked improvement in oral health observed during those years.

As strengths of the study, the authors highlight the longitudinal analysis of inequalities using nationally comprehensive data, which ensures representativeness. Another important aspect is the study’s contribution to the global understanding of oral health data, especially by using the DMFT index, a complete and consolidated metric, but which, due to the high cost and complexity of the clinical studies necessary for its territorial production, is being progressively replaced by simpler indicators. Therefore, the use of DMFT in this study should be even more valued, given its rigor and the scarcity of productions of this nature. As a limitation, the authors acknowledge the self-reported collection of socioeconomic variables, which may introduce a risk of information bias; however, it is expected that this is randomly distributed among the different strata, in addition to the parsimonious analysis that did not include other determinants that imply this scenario, such as race/ethnicity and region of residence.

An analysis of the oral health of Brazilian adolescents over the past 20 years suggests that, although there has been a general improvement in oral health indicators, inequalities between different social groups remain. More socially vulnerable populations have not experienced the same advances observed among adolescents in better socioeconomic conditions, indicating that current public policies, while effective in overall improvement, have not been sufficient to reduce social barriers and, in some cases, may have exacerbated disparities, such as those associated to education level.

To mitigate this scenario, future policies should adopt intersectoral approaches that integrate oral health actions with education, basic sanitation, and social policy strategies, modulating the structural determinants that maintain vulnerability. In addition, eminently preventive programs should be developed for different age groups, with priority given to historically disadvantaged groups, ensuring an impact based on equity.
