# Supplementary material for: Trends in Socioeconomic Inequalities in the Distribution of Dental Caries Among Brazilian Adolescents, 2003–2023
Source: Rev Bras Epidemiol. 2026 Jul 31;29(Suppl 1):e260017supl1. doi: 10.1590/1980-549720260017.supl.1 (PMC13427310; doi:10.1590/1980-549720260017.supl.1)
Supplement: Translation

# Translation: Tendências das desigualdades socioeconômicas na distribuição da cárie dentária em adolescentes brasileiros entre 2003 e 2023

**DOI:** 10.1590/1980-549720260017.supl.1.2

**Authors:** Sophia Queiroz Marques dos Santos, Angelo Giuseppe Roncalli

**Affiliations:** I Universidade Federal do Rio Grande do Norte, Programa de Pós-Graduação em Saúde Coletiva – Natal (RN), Brasil.

**Keywords:** Mensuração das desigualdades em saúde, Saúde bucal, Inquéritos epidemiológicos, Cárie dentária

## Abstract

**Objetivo:**

Analisar as tendências das desigualdades socioeconômicas na distribuição da cárie dentária em adolescentes nos anos de 2003, 2010 e 2023.

**Métodos:**

Estudo observacional longitudinal, com dados de inquéritos nacionais de saúde bucal. Foram analisados o índice de Dentes Cariados, Perdidos e Obturados (CPO-D) e o número de dentes cariados, estratificados por renda e escolaridade. As análises consideraram o desenho amostral complexo e incluíram os índices de desigualdade absoluta (*Slope Index of Inequality* – SII) e relativa (*Relative Index of Inequality* – RII). A significância estatística foi avaliada ao nível de 95% de confiança (p < 0,05).

**Resultados:**

Observou-se redução global do CPO-D e do número de dentes cariados em todos os estratos socioeconômicos ao longo do período, indicando melhora geral das condições de saúde bucal. Entretanto, persistiram desigualdades marcantes entre os grupos, com manutenção do gradiente social, no qual adolescentes com menor renda e escolaridade apresentaram piores indicadores. O SII indicou estabilidade das diferenças absolutas entre os extremos sociais, enquanto o RII evidenciou variações ao longo do tempo, sugerindo mudanças proporcionais nas desigualdades. Para o CPO-D, observou-se aumento das desigualdades segundo escolaridade e discreta redução em relação à renda; para dentes perdidos, houve redução das desigualdades nos anos mais recentes.

**Conclusão:**

Apesar da melhora geral dos indicadores de cárie, as desigualdades socioeconômicas em saúde bucal entre adolescentes persistem, refletindo determinantes sociais estruturais. Os achados reforçam a necessidade de políticas públicas direcionadas à redução das iniquidades e à promoção da equidade em saúde bucal.

## INTRODUÇÃO

Os determinantes sociais da saúde refletem as condições de vida e os arranjos sociais que influenciam o desenvolvimento de doenças^1^. Esses determinantes, por sua vez, funcionam como um recorte específico em um conjunto maior, com o objetivo de identificar como diferentes grupos sociais apresentam desfechos de saúde distintos. A análise das desigualdades revela o gradiente social de saúde, um padrão hierarquizado na distribuição das doenças, que sugere que variações no status socioeconômico impactam diretamente a susceptibilidade às condições de saúde^2^.

Para ilustrar esse fenômeno, podemos considerar a Insegurança Alimentar e Nutricional (Isan), tradicionalmente associada à desnutrição. Hoje, após mudanças significativas no mercado e no “fazer social” – conceituado por Max Weber –, o sobrepeso e a obesidade tornaram-se novos marcadores de vulnerabilidade, ampliando o conceito de Isan, que agora abrange também formas de subnutrição^3^. Apesar das transformações comportamentais e da introdução de novas óticas para a análise de agravos, o gradiente social sobre o desfecho é tão fortemente atuante que populações específicas continuam a experimentar as piores condições de saúde. Situação semelhante é observada na saúde bucal.

No contexto da saúde bucal, um tema crucial é o custo para garantir a efetividade dos serviços no sistema público de saúde. Estamos falando não apenas do financiamento necessário para o tratamento de lesões já presentes, mas também da previsão de gastos com lesões iminentes. O subfinanciamento do sistema contribui diretamente para a perpetuação das desigualdades e revela importantes marcadores sociais, uma vez que o custo do tratamento para indivíduos em condições socioeconômicas mais baixas está relacionado à negligência histórica e social. Isso resulta em maior prevalência ou agravamento de lesões, ampliando custos – ainda não supridos totalmente^4,5^. Na prática, o acesso igualitário à saúde bucal é um desafio, pois diversos fatores individuais, organizacionais e sociais dificultam o cumprimento desse princípio^6^. O desenvolvimento da doença frequentemente ultrapassa a capacidade de tratamento, que avança de forma lenta.

Em estudo realizado em 2015 por Roncalli et al., foi observado que o declínio de cárie em adolescentes brasileiros entre 2003 e 2010 se deu de forma desigual e que os indicadores de desigualdade analisados nesses dois períodos apontaram para um agravamento das diferenças socioeconômicas^7^. De forma similar, uma investigação comparada da Pesquisa Nacional de Saúde do Escolar (PeNSE) 2009, 2012 e 2015 mostrou que a prevalência de dor dentária aumentou de 17,5% para 21,8% entre os anos extremos, com adolescentes de mães com menor escolaridade apresentando consistentemente maiores níveis de dor dentária^8^. Uma análise mais atual para adolescentes de 12 anos em 2003, 2010 e 2023 indicou uma redução geral da experiência de cárie, porém com persistência de desigualdades regionais, onde o pior estrato possuía desfecho cerca de quatro vezes maior que a média^9^.

A polarização na distribuição das doenças bucais não constitui um fenômeno recente, ainda assim, seu monitoramento contínuo é imprescindível para identificar padrões distributivos e subsidiar estratégias verdadeiramente capazes de mitigar a persistência da desigualdade. Nesse sentido, este estudo busca dar continuidade à investigação das tendências de desigualdades socioeconômicas na distribuição da cárie em adolescentes entre os anos 2003, 2010 e 2023.

## MÉTODOS

Trata-se de um estudo observacional longitudinal, desenvolvido com dados secundários provenientes dos três últimos inquéritos nacionais de saúde bucal, conhecidos como Projeto SB Brasil (2003, 2010 e 2023)^10–12^, aprovados pelos respectivos Comitês de Ética em Pesquisa (processos 25000.009632/00-51, n.° 1.356, n.° 15.498 e n.° 4.823.054). Os levantamentos utilizaram metodologias semelhantes, sem prejuízo à representatividade e comparabilidade dos dados. Os principais desfechos investigados incluíram cárie dentária, condição periodontal, oclusopatias e necessidade e uso de próteses dentárias. Informações detalhadas sobre a metodologia do Projeto SB Brasil podem ser encontradas em publicações específicas, especialmente nos trabalhos de Roncalli & Souza^13,14^.

A amostra foi composta por adolescentes de 15 a 19 anos, totalizando 16.833 indivíduos em 2003, 5.445 em 2010 e 8.054 em 2023. Essa faixa etária foi selecionada pela disponibilidade de dados socioeconômicos individuais e pela menor variabilidade das taxas de cárie, favorecendo análises de associação.

### Variáveis

Os desfechos foram o índice de Dentes Cariados, Perdidos e Obturados (CPO-D) e o número de dentes cariados (componente “C”). As variáveis independentes foram renda familiar mensal e nível educacional, disponíveis nos questionários das três pesquisas. A renda foi mensurada como a renda total familiar no mês anterior à entrevista, registrada em valores absolutos em 2003 e 2023, e em classes em 2010. Para padronização, a renda foi convertida em salários mínimos vigentes em cada ano (2003: R$ 210; 2010: R$ 510; 2023: R$ 1.320) e categorizada em 4 estratos.

A escolaridade foi expressa em anos de estudo e ajustada por idade, utilizando o indicador de distorção idade-série, amplamente empregado na avaliação de políticas educacionais. A variável foi categorizada em quatro estratos (acima do nível ideal, no nível ideal, 1-2 anos de atraso e ≥ 3 anos de atraso), seguindo a estratégia adotada por Roncalli et al.^7^.

### Análise dos dados

Inicialmente, realizou-se análise descritiva das médias do índice CPO-D e do número de dentes cariados, com seus respectivos intervalos de confiança de 95%, segundo estratos socioeconômicos e anos dos inquéritos (2003, 2010 e 2023). Considerando que os três inquéritos utilizaram desenho amostral complexo^14^, todas as estimativas incorporaram pesos amostrais e efeito de delineamento, incluindo as Unidades Primárias de Amostragem (UPA) como *clusters*.

### Conceituação e interpretação dos índices de desigualdade

A desigualdade na distribuição da cárie dentária foi mensurada por meio do Índice de Inclinação da Desigualdade (*Slope Index of Inequality* – SII) e do Índice Relativo de Desigualdade (*Relative Index of Inequality* – RII), calculados para cada desfecho, variável socioeconômica e ano analisado. Também foi estimado o efeito de interação entre os inquéritos para avaliar mudanças nos indicadores de desigualdade entre 2003, 2010 e 2023.

O SII e o RII foram estimados por Modelos Lineares Generalizados (GLM) com distribuição de Poisson, conforme recomendações de Mackenbach & Kunst (1997)^15^ e Barros & Victora (2013)^16^. As variáveis independentes foram transformadas em escores ridit, com base na distribuição proporcional da amostra em cada categoria. O SII representa a diferença absoluta entre os extremos da hierarquia social, enquanto o RII expressa a razão relativa entre esses grupos. As análises consideraram pesos amostrais e efeito de *cluster* por meio do comando *svy* do software *Stata*.

Gráficos em *equiplots* foram utilizados para ilustrar as desigualdades, conforme metodologia proposta pelo Centro Internacional de Equidade em Saúde da Universidade Federal de Pelotas (UFPel). Visualmente, maiores distâncias entre os pontos ao longo da escala socioeconômica indicam maior magnitude de desigualdade (https://equidade.org/pt/home).

### Declaração de disponibilidade de dados

Todo o conjunto de dados que dá suporte aos resultados deste estudo foi disponibilizado no SciELO Data (https://doi.org/10.48331/SCIELODATA.HINAG2) e pode ser acessado em https://www.gov.br/saude/pt-br/composicao/saps/brasil-sorridente/sb-brasil/dados.

## RESULTADOS

A Tabela 1 e a Figura 1 apresentam os resultados da análise descritiva que compara o CPO-D e o número de dentes cariados para cada estrato das variáveis socioeconômicas (renda e nível educacional) e para os anos pesquisados.

**Tabela 1:** CPO-D médio (valor absoluto) e média de dentes cariados (valor relativo à média geral) em adolescentes de 15–19 anos, segundo variáveis socioeconômicas e ano da pesquisa.

	n	CPO-D		Dentes cariados	
		Média	IC95%	Média	IC95%
Renda familiar (salários mínimos – SM)					
2003					
Maior que 5 SM	5.298	5,29	4,85–5,74	1,81	1,55–2,08
3 a 5 SM	3.724	5,65	5,19–6,10	2,54	2,28–2,80
1 a 2 SM	3.546	5,98	5,47–6,50	3,08	2,78–3,37
Menor que 1 SM	3.955	5,29	4,9–5,68	2,88	2,56–3,2
2010					
Maior que 5 SM	665	3,04	2,36–3,72	0,65	0,44–0,86
3 a 5 SM	920	3,61	2,99–4,24	1,06	0,80–1,32
1 a 2 SM	2.621	4,47	4,04–4,91	1,85	1,55–2,15
Menor que 1 SM	846	5,36	4,51–6,21	2,86	2,19–3,52
2023					
Maior que 5 SM	343	2,77	1,45–4,09	0,35	0,16–0,53
3 a 5 SM	689	1,96	1,15–2,76	0,60	0,33–0,86
1 a 2 SM	2.563	3,57	3,01–4,12	1,54	1,25–1,82
Menor que 1 SM	1.416	3,74	3,15–4,33	2,26	1,86–2,66
Nível educacional (distorção idade-série)					
2003					
Acima	3.054	4,94	4,49–5,39	1,60	1,34–1,85
Nível ideal	5.451	5,21	4,79–5,63	1,93	1,69–2,17
1-2 anos	3.509	5,74	5,32–6,16	3,00	2,72–3,28
3 ou mais anos	4.008	6,43	6,03–6,84	3,59	3,26–3,91
2010					
Acima	1.398	3,23	2,76–3,70	1,04	0,79–1,28
Nível ideal	2.044	4,24	3,66–4,82	1,54	1,25–1,84
1-2 anos	926	4,81	4,08–5,53	2,40	1,09–2,90
3 ou mais anos	662	6,07	5,24–6,89	3,06	2,44–3,69
2023					
Acima	3.390	2,66	2,20–3,12	1,12	0,88–1,35
Nível ideal	3.507	3,87	3,40–4,35	1,71	1,46–1,95
1–2 anos	508	4,84	3,90–5,77	2,41	1,74–3,08
3 ou mais anos	200	5,61	4,11–7,11	3,51	2,44–4,58

**Figura 1:** Equiplots ilustrando a distribuição do CPO-D em relação a renda (A) e nível educacional (B) e do número de dentes cariados em relação a renda (C) e nível educacional (D), para os três anos estudados. Fonte: Elaboração dos autores com base nos bancos de dados do SB Brasil 2003, 2010 e 2023.

Observa-se redução no CPO-D médio entre 2003 e 2023 em todos os estratos de renda e nível educacional. Na análise do grau de desigualdade, espera-se observar um gradiente em que os estratos mais favorecidos terão sempre valores mais favoráveis, que pioram à medida que os estratos menos favorecidos também pioram, expressando efeito dose-resposta.

Esse padrão está presente em praticamente todas as análises, exceto para o CPO-D em relação à renda em 2003 (mesmos valores para o primeiro e quarto estratos) e para o número de dentes cariados em relação à renda no mesmo ano (inversão entre o primeiro e segundo estratos). Contudo, em ambos os casos, os valores são próximos e não há diferença significativa, pela análise dos intervalos de confiança.

Pode-se inferir que há efeito dose-resposta para CPO-D e dentes cariados em relação a renda e nível educacional, expressando, na prática, a desigualdade na distribuição da doença. Deve-se observar, no entanto, a magnitude desse efeito e se ele se altera ao longo dos anos. Na análise visual, observa-se se a distância entre os círculos se altera entre os anos estudados. Para CPO-D, há maior aprofundamento da desigualdade entre 2003 e 2010, seguido de estabilidade entre 2010 e 2023, tanto para renda quanto para nível educacional. Para dentes cariados, o perfil é similar, com ressalva para a relação com nível educacional, que apresenta padrão de desigualdade que tendencia uma melhora, mas é relativamente semelhante entre os anos.

De modo geral, a análise das desigualdades em saúde bucal revelou que, para todos os desfechos avaliados, persistem diferenças marcantes entre os extremos de renda e escolaridade, evidenciadas pelos índices SII e RII. O SII, medido pela diferença no desfecho entre os grupos mais favorecidos e menos favorecidos, apresentou pouca variação estatisticamente significativa ao longo do período, indicando que, em termos absolutos, a distância entre os extremos manteve-se estável. Em contrapartida, o RII, que expressa a razão entre os extremos, apresentou alterações significativas em diversos casos, sugerindo que a força proporcional da desigualdade sofreu modificações ao longo dos anos (Tabela 2).

**Tabela 2:** Índice de desigualdade absoluto (SII) e relativo (RII) para a média do CPO-D e dos dentes cariados. O valor de p indica a presença de variações significativas entre os anos.

	SII (IC95%)				RII (IC95%)			
	2003	2010	2023	p valor	2003	2010	2023	p valor
CPO-D								
Renda familiar (salários mínimos)	0,38 (-0,42–1,17)	2,66 (1,57–3,75)	1,91 (0,41–3,40)	**0,051**	1,06 (0,92–1,22)	1,87 (1,44–2,42)	1,70 (1,10–2,61)	**0,006**
Educação (distorção idade-série)	1,46 (0,86–2,07)	2,17 (1,20–3,14)	2,26 (1,25–3,26)	0,301	1,31 (1,17–1,48)	1,67 (1,34–2,09)	1,79 (1,38–2,33)	**0,038**
Dentes cariados								
Renda familiar (salários mínimos)	1,86 (1,35–2,37)	2,48 (1,84–3,11)	2,42 (1,86–2,98)	0,163	2,04 (1,68–2,48)	4,67 (3,16–6,92)	4,49 (2,92–6,94)	**<0,001**
Educação (distorção idade-série)	2,14 (1,7–2,52)	1,52 (1,00–2,03)	1,09 (0,52–1,66)	0,209	2,69 (2,19–3,31)	2,51 (1,81–3,48)	1,91 (1,37–2,64)	**<0,001**

No caso do RII para CPO-D, observa-se inicialmente aumento, seguido de redução das desigualdades relacionadas à renda. Entretanto, a análise segundo escolaridade indica distanciamento crescente entre os grupos, evidenciando impactos distintos de fatores sociais sobre a saúde bucal. Em relação ao número de dentes cariados, o comportamento segue padrão semelhante à renda, com tendência de redução das desigualdades ao longo do tempo (Tabela 2). Isso sugere que, embora os adolescentes tenham ampliado o acesso a serviços odontológicos e reduzido dentes com cárie, tais avanços ainda não se refletem em diminuição efetiva da experiência de cárie.

## DISCUSSÃO

É possível observar uma mudança desigual no padrão de adoecimento, com um descompasso entre o declínio da média de dentes cariados e o CPO-D. A continuidade da experiência de cárie mostra uma regressão lenta, com um padrão de diminuição do componente cariado ao longo do tempo, mais acentuada em grupos mais favorecidos. A distância entre esses grupos é influenciada principalmente pela renda e pela situação escolar. o índice de desigualdade absoluta não mostrou diferença significativa entre os três períodos, indicando que a desigualdade permanece ao longo do tempo.

No entanto, o índice relativo mostrou mudança significativa, destacando as diferenças na probabilidade de adoecer. Em 2003, a experiência de cárie no grupo de menor renda foi 6% maior que no grupo de maior renda, aumentando expressivamente para 87% em 2010 e reduzindo sutilmente para 70% em 2023 (p=0,006), evidenciando uma desigualdade persistente – e um movimento semelhante ocorre para os dentes cariados. Para o nível educacional, a experiência de cárie no grupo menos favorecido era 31% em 2003, aumentando para 67% em 2010 e 79% em 2023 (p=0,038); a desigualdade entre os níveis educacionais se acentuou no CPO-D, mas o mesmo movimento não é observado para os dentes cariados, com diminuição ao longo dos anos. Esses resultados são fundamentais para orientar a formulação de estratégias no âmbito da Política Nacional de Saúde Bucal (PNSB), com foco na população estudada, visando à redução das desigualdades em saúde bucal.

Recortes a respeito da distribuição socialmente desigual de doenças crônicas são comuns, ademais, raramente se dispõe de uma comparação ao longo do tempo, principalmente para as doenças bucais. Um estudo que associava comportamentos individuais ao desenvolvimento dessas doenças revelou que, independentemente das práticas de saúde, existia uma lacuna entre grupos, resultado de fatores sociais mais profundos^17^. De forma complementar, uma análise de custos dos tratamentos bucais em seis países (Brasil, França, Alemanha, Indonésia, Itália e Reino Unido) revelou que, para a maioria deles, o maior custo estava centrado na população do estrato social mais vulnerável, exceto para a Indonésia^18^, reafirmando uma distribuição heterogênea e socialmente graduada.

Esses achados revelam que sociedades com comportamentos, sistemas de saúde e condições de vida diferentes ainda refletem em diferentes níveis o arranjo distributivo da doença. Buscando detalhar o cenário local, a pesquisa condensa um questionário socioeconômico aos achados clínicos, de onde foi possível extrair as variáveis como renda e escolaridade, essenciais na criação de um modelo de determinação. Ademais, a evolução das políticas e sua expressão na sociedade são de extrema relevância para os resultados da pesquisa, compondo o último nível de um modelo de determinação. Conquistas relacionadas aos direitos sociais começaram a se desenvolver no Brasil na década de 1990, enquanto os anos 2000 foram marcados por tentativas de operacionalização e as décadas posteriores simbolizam uma maior efetividade do serviço, justificando a grande evolução da situação de saúde bucal notável nos dados de 2003 e 2010^19^.

As discrepâncias entre os anos seguintes, além de bastante inferiores, nem sempre culminam em redução. A avaliação desses serviços pode ser um dos elementos-chave para justificar esse cenário. A PNSB, que completou 20 anos em 2024, foi fundamental na tentativa de aumentar a cobertura e melhorar o acesso à saúde bucal^20^. Além disso, existem disparidades na utilização dos serviços, tendo em vista que a população negra das regiões Norte e Nordeste, de baixa condição econômica e escolaridade, tem maior chance de acompanhamento odontológico irregular e de realizar procedimentos cirúrgicos ou de urgência^21^, indicando que a implementação da política não foi suficiente para mitigar desigualdades no acesso a consultas odontológicas^22^. O estudo de Freire et al. demonstrou que o “não acesso” esteve frequentemente associado a fatores municipais, como maior desigualdade, fatores organizacionais, como menor cobertura e tempo de deslocamento até a unidade, além dos fatores individuais como idade e renda^23^.

O problema, no entanto, pode se estender à distribuição dos serviços e oferta de profissionais. É possível atrelar a maior prevalência de equipes de saúde bucal a cidades com Índice de Desenvolvimento Humano (IDH) muito alto, situadas na região Sudeste e com cobertura menor que 50%^24^, enquanto a mão de obra também apresenta maior proporção a cada 10 mil habitantes nas regiões Sul e Sudeste^25^. Apesar de as políticas públicas de saúde bucal melhorarem o panorama, elas não eliminaram as desigualdades de acesso e cuidado, especialmente entre os grupos mais vulneráveis, algo que é nitidamente evidenciado pelos resultados do estudo.

É importante, também, refletir sobre como a faixa etária e os contextos históricos influenciam os resultados. Considerando que crianças de 5 anos em 2010 tinham, em 2023, 18 anos, ressalta-se que nasceram em uma época marcada pela implementação da PNSB e vivenciaram uma realidade totalmente diferente quando comparadas às crianças de 18 anos em 2010, que aos 11 (2003) anos sofriam somente o impacto do gradiente social. É inegável que a política pública em certa medida atenua as desigualdades para todos os grupos, mas de maneiras diferentes^26^. Crianças mais novas, mesmo as nascidas após a implementação da PNSB, ainda enfrentam barreiras sociais significativas quando pertencem a estratos socioeconômicos mais baixos – desafios que muitas vezes extrapolam a eficácia da política^27^. Assim, a redução das doenças bucais não ocorre de maneira homogênea entre os grupos. Nos mais vulneráveis, mesmo com as políticas públicas, os avanços na saúde bucal tendem a ser mais lentos e desiguais; e o contrário também é verdadeiro. Paradoxalmente, isso pode acabar ampliando as disparidades existentes em vez de diminuí-las.

O impacto significativo dos determinantes como a educação e a renda na saúde bucal tem sido amplamente estudado na literatura internacional^28,29^. Indivíduos com menor nível educacional tendem a valorizar menos as práticas preventivas de saúde bucal, o que, somado à falta de acesso a informações adequadas, aumenta a prevalência de cáries e outras doenças bucais^28^. Da mesma forma, a renda mais baixa está diretamente relacionada à dificuldade de acesso a serviços de saúde bucal de qualidade, incluindo consultas regulares e tratamentos preventivos, além de estarem frequentemente associados a dietas mais ricas em açúcares e alimentos processados, contribuindo para o agravamento de doenças bucais^29^. Não obstante, a interação desses determinantes potencializa o cenário de doença.

Embora a escolaridade seja frequentemente associada a maior acesso à informação e a comportamentos preventivos, como reflete a Teoria da Incorporação Social^30^, que enfatiza repetições comportamentais determinadas socialmente, sua relação com indicadores cumulativos como o CPO-D é mais complexa, algo nitidamente captado neste estudo. Mudanças no nível educacional ou a qualidade da educação formal acessada não necessariamente se traduzem em redução imediata da experiência acumulada de cárie, uma vez que esse indicador reflete exposições e padrões de cuidado construídos ao longo do curso de vida. Nesse sentido, a escolaridade atua como marcador estrutural de trajetórias sociais e de acesso historicamente desigual aos serviços de saúde bucal, influenciando mais fortemente o tipo de cuidado recebido (preventivo ou mutilador) do que o curso de atividade atual da doença. Isso tem forte relação com a Epidemiologia do Curso da Vida^31^, incitando que exposições iniciais, sejam elas positivas ou negativas, reverberam no indivíduo ao longo dos anos. O citado ajuda a explicar por que a redução das desigualdades foi mais evidente segundo a renda e no componente ativo da doença, enquanto as desigualdades relativas à escolaridade persistiram e se intensificaram no CPO-D.

Em países como o Reino Unido, com políticas públicas como o Sistema Nacional de Saúde (NHS, na sigla em inglês), houve avanços importantes na redução das desigualdades em saúde bucal, oferecendo tratamentos gratuitos ou a preços acessíveis para diferentes faixas etárias. No entanto, mesmo com essas políticas, persistem disparidades significativas nos resultados de saúde bucal entre diferentes grupos socioeconômicos^32^. Uma pesquisa realizada pela Organização Mundial da Saúde (OMS) em 2017 destaca que, apesar dos esforços de prevenção, os determinantes proximais continuam sendo os maiores preditores das condições de saúde bucal, com as populações mais desfavorecidas, especialmente as de baixa renda e escolaridade, sofrendo de maneira mais acentuada com as doenças bucais^33^.

O caráter multifatorial das doenças bucais exige uma resposta integrada e abrangente das políticas públicas, que deve considerar a complexidade das condições sociais e econômicas^34^. Para combater efetivamente as desigualdades, é necessário ir além da promoção de cuidados (epidemiologicamente determinada, corretamente financiada e intersetorialmente integrada), extrapolando para o avanço e a integração de demais políticas públicas. As políticas de saúde bucal muitas vezes atuam isoladamente, sem transversalidade, mas, quando combinadas com políticas de saneamento básico e alimentação, os efeitos podem ser multiplicados, podendo ser mais eficazes em uma população na qual as condições sanitárias são adequadas e os hábitos alimentares são mais equilibrados^35^. O fortalecimento dessas políticas simultaneamente ajudaria a criar um ambiente mais favorável à saúde bucal. Como dito anteriormente, o período dos anos 2000/2010 foi marcado por foco em políticas de saúde pública e redução da pobreza no Brasil^36^, o que pode ter contribuído para a melhoria acentuada da saúde bucal observada entre os anos.

Como pontos fortes do estudo, os autores destacam a análise longitudinal das desigualdades utilizando dados de abrangência nacional, o que garante a representatividade. Outro aspecto importante é a contribuição do estudo para a leitura global dos dados de saúde bucal, especialmente ao utilizar o índice CPO-D, uma métrica completa e consolidada, mas que, devido ao alto custo e à complexidade dos estudos clínicos necessários para sua produção territorial, está sendo progressivamente substituída por indicadores mais simples. Portanto, o uso do CPO-D neste estudo deve ser ainda mais valorizado, dado o seu rigor e a escassez de produções dessa natureza. Como limitação, os autores reconhecem a coleta autorrelatada das variáveis socioeconômicas, o que pode introduzir um risco de viés de informação; contudo, espera-se que este esteja aleatoriamente distribuído entre os diferentes estratos, além da análise parcimoniosa que não incluiu outros determinantes que implicam esse cenário, como cor/raça e região de moradia.

A análise da saúde bucal de adolescentes brasileiros ao longo dos últimos 20 anos sugere que, embora tenha ocorrido uma melhora geral nos indicadores de saúde bucal, as desigualdades entre diferentes grupos sociais permanecem. Populações mais vulneráveis socialmente não experimentaram os mesmos avanços observados entre adolescentes em melhores condições socioeconômicas, indicando que as políticas públicas atuais, embora eficazes na melhoria geral, não foram suficientes para reduzir as barreiras sociais e, em certos casos, podem ter exacerbado disparidades, como as associadas à escolaridade. Para mitigar esse cenário, futuras políticas devem adotar abordagens intersetoriais que integrem ações de saúde bucal com estratégias de educação, saneamento básico e políticas sociais, modulando os determinantes estruturais que mantêm a vulnerabilidade. Além disso, programas eminentemente preventivos devem ser desenvolvidos para diferentes faixas etárias, com direção prioritária a grupos historicamente desfavorecidos, garantindo impacto pautado em equidade.

## COMITÊ DE ÉTICA EM PESQUISA:

O estudo não utiliza dados primários, porquanto não necessitou de aprovação em comitê de ética.
